# Song sparrows *Melospiza melodia* have a home-field advantage in defending against sympatric malarial parasites

**DOI:** 10.1098/rsos.160216

**Published:** 2016-08-10

**Authors:** Yanina Sarquis-Adamson, Elizabeth A. MacDougall-Shackleton

**Affiliations:** Department of Biology, University of Western Ontario, London, Ontario, Canada

**Keywords:** cross-infection experiment, host–parasite interactions, local adaptation, haematozoa, *Melospiza melodia*, *Plasmodium*

## Abstract

Hosts and parasites interact on both evolutionary and ecological timescales. The outcome of these interactions, specifically whether hosts are more resistant to their local parasites (sympatric) than to parasites from another location (allopatric), is likely to affect the spread of infectious disease and the fitness consequences of host dispersal. We conducted a cross-infection experiment to determine whether song sparrows (*Melospiza melodia*) have an advantage in dealing with sympatric parasites. We captured birds from two breeding sites 437 km apart, and inoculated them with avian malaria (*Plasmodium* spp.) cultured either from their capture site or from the other site. Infection risk was lower for birds exposed to sympatric than to allopatric *Plasmodium* lineages, suggesting that song sparrows may have a home-field advantage in defending against local parasite strains. This pattern was more pronounced at one capture site than at the other, consistent with mosaic models of host–parasite interactions. Home-field advantage may arise from evolutionary processes, whereby host populations become adapted to their local parasites, and/or from ecological interactions, whereby host individuals develop resistance to the local parasites through previous immune exposure. Our findings suggest that greater susceptibility to novel parasites may represent a fitness consequence of natal dispersal.

## 1. Introduction

Parasites and their hosts are key features of one another's environments, interacting on both evolutionary timescales (through local arms races) and ecological timescales (through parasite effects on host fitness and the development of resistance within the lifetime of host individuals). The outcome of these interactions, i.e. whether parasites are better able to infect hosts from the same area (sympatric) than hosts from a different area (allopatric) or whether hosts are more resistant to infection by sympatric than allopatric parasites, can have important consequences for host diversification [1]. For example, if hosts are more resistant to allopatric than sympatric parasites, selection may promote natal dispersal as a means of escaping sympatric parasites and contribute to the success of invasive species [2]. Conversely, if hosts are more resistant to sympatric than allopatric parasites, then the risk of encountering allopatric parasites may represent a cost of dispersal [3].

The reciprocal selection forces that hosts and parasites impose on one another can generate local cycles of antagonistic coevolution [4]. Selection on parasites favours the ability to exploit locally common host genotypes, potentially resulting in parasite local adaptation, whereby parasites are better able to infect sympatric than allopatric hosts [5]. At the same time, selection on hosts favours resistance to locally common parasite strains, potentially resulting in host local adaptation, i.e. greater resistance to sympatric than allopatric parasites [6]. In general, larger population sizes and shorter generation times in parasites than hosts are thought to provide parasites with an evolutionary advantage [7]. However, host–parasite coevolution can also be influenced by factors such as relative rates of gene flow, virulence and host specificity of the parasite [8]. Thus, although parasite local adaptation appears to be common, in some systems hosts show local adaptation to parasites, and others show a mosaic pattern such that the outcome of host–parasite interactions varies across populations [9].

In addition to evolutionary interactions between hosts and parasites, the exposure history of individual hosts can also influence their resistance to particular parasites. In jawed vertebrates, the acquired immune response facilitates the development of immunological memory. Following exposure to antigens, particularly during early life, individual hosts develop antigen-specific lymphocyte lines and antibodies [10], essentially vaccinating them against repeated encounters with the same antigen [11]. Such immunological memory permits a rapid and efficient secondary response to pathogens that an individual host has encountered previously. In humans, for example, exposure to malaria parasites (*Plasmodium falciparum*) during early life confers protection against subsequent infection by similar strains [12]. Thus, vertebrate hosts may have a home-field advantage over sympatric parasites owing to population-level evolutionary processes (i.e. host local adaptation) and/or individual-level ecological processes (i.e. immunological memory). Changing ranges of parasites and their vectors, associated with a changing climate [13], emphasize the importance of predicting the outcome of evolutionary and ecological interactions between hosts and parasites.

Interactions between birds and their parasites are of keen interest from a diversity of perspectives, including speciation [1], conservation [14], sexual selection [15] and zoonotic disease [16]. Correlative evidence from natural populations suggests that at least in some cases, birds may be more resistant to sympatric than to allopatric parasites. Male white-crowned sparrows (*Zonotrichia leucophrys*) that sing local songs, and thus presumed to be of local origin, have lower haematozoan parasite loads than males singing non-local songs and thus presumed to be immigrants [17]. Similar patterns have been observed in song sparrows (*Melospiza melodia*) [18]. As well, female barn swallows (*Hirundo rustica*) that remain to breed in their natal colony have lower ectoparasite (louse fly) infestations than do females that disperse to breed outside their natal colony [19]. Lower parasite loads in philopatric than in dispersing individuals suggest that philopatry may confer home-field advantage in dealing with the local parasite fauna. However, other potential explanations for this pattern cannot be excluded. Philopatric and dispersing individuals may differ in morphology, behaviour or other traits that may affect susceptibility to parasites [19,20]. Thus, conclusively determining whether low parasite loads in philopatric birds reflect home-field advantage or simply differences in quality between philopatric and dispersing hosts requires an experimental approach.

We conducted a reciprocal cross-infection experiment to test whether migratory song sparrows have an advantage in defending against their local malarial parasites (*Plasmodium* spp.), or conversely whether parasites in this system have an advantage in infecting their local hosts. We captured sparrows from two different breeding sites, identified locally confined *Plasmodium* lineages in each, and assessed resistance to sympatric versus allopatric lineages by measuring infection status and severity. If parasites have a home-field advantage in infecting their local hosts, then *Plasmodium* spp. cultured from one site should be better able to infect and/or proliferate in its sympatric than allopatric hosts. Conversely, if hosts have a home-field advantage in resisting their local parasites, then birds should be more resistant to sympatric than to allopatric *Plasmodium* lineages.

## 2. Material and methods

### 2.1. Study system: hosts and parasites

We captured song sparrows from two breeding locations separated by 437 km: an eastern site at Newboro, Ontario, Canada (44.633°N, 76.330°W) and a western site at London, Ontario, Canada (43.008°N, 81.291°W). Song sparrows in these areas are seasonally migratory, and show moderate natal philopatry combined with high adult philopatry. At the eastern site, where we have studied breeding biology since 2002, approximately 10% of breeding adults each year were first banded as nestlings on the site; and nearly 50% of breeding adults (presumably all those surviving the winter) return to the site the following year [21].

The most commonly observed blood-borne parasites in these birds are *Plasmodium* spp. (Apicomplexa). These intracellular parasites are normally transmitted between avian hosts by *Culex* mosquitoes [22]. However, because asexual reproduction (schizogony) occurs in circulating erythrocytes as well as in fixed tissues of the vertebrate host, *Plasmodium* spp. are highly amenable to infectivity experiments, because infections can be transmitted to new hosts through inoculation with infected blood [23,24].

Using mist nets and seed-baited Potter traps, we captured 16 adult song sparrows (eight males, eight females) from the eastern site, and 18 adult song sparrows (14 males, four females) from the western site. Seven birds (four males, three females) were captured in October 2011, and 27 (18 males, nine females) between July and September 2012. We transported birds in individual cages to the Advanced Facility for Avian Research at the University of Western Ontario. Birds were given ad libitum access to food and water and maintained in individual cages, in indoor rooms free of insect vectors, until the experiment began in October 2012.

### 2.2. Characterizing naturally occurring infections

To identify birds that were already naturally infected with *Plasmodium* spp., we collected a small (25 µl) blood sample via brachial venipuncture of each individual. A drop of this sample was used to prepare a thin-film blood smear. Smears were air-dried, fixed in 100% methanol, treated with Wright–Giemsa stain and examined under a light microscope with a 100× objective. For each bird, we scanned 10 000 erythrocytes and noted the number of cells containing one or more haematozoan parasites.

From the remainder of the blood sample, we extracted DNA using an ammonium-acetate-based protocol, then used a two-stage, nested PCR approach to amplify parasite cytochrome *b* [25]. The first stage used primers HAEMNFI and HAEMNR3 [25] to amplify an initial 617-bp fragment of cytochrome *b* of genera *Plasmodium*, *Haemoproteus* and *Leucocytozoon*. The second stage used 1 µl of product from the initial PCR as template, along with the internally nested primers HAEMF and HAEMR2 [26] to amplify 527 bp of cytochrome *b* of *Haemoproteus* and *Plasmodium*. PCR was conducted in 25 µl volumes using conditions described elsewhere [25]. Second-round PCR products were run at 100 V for 90 min on 2% agarose gels, stained with ethidium bromide, and visualized under UV light. Bands of the expected product size were excised and purified using a Gel/PCR DNA extraction kit (FroggaBio, North York) then sequenced from the 5′ end with primer HAEMF on an ABI 3730 Genetic Analyser (Applied Biosystems) at the London Regional Genomics Centre. Sequences were aligned using ClustalW, trimmed to 476 bp, and identified to genus (i.e. *Plasmodium* or *Haemoproteus*) using the basic local alignment search tool implemented in GenBank. We observed only one instance of double peaks on electropherograms, suggesting that co-infections were rare, although see [27].

### 2.3. Identifying locally confined lineages

Additional surveys of song sparrows and their *Plasmodium* parasites were not possible throughout western Ontario. Thus, we cannot exclude the possibility that parasite lineages detected only at the eastern site might also occur (at least at low frequency) at the western site. However, we conducted wider sampling of song sparrows throughout eastern Ontario to identify which, if any, lineages detected at the western site were absent from the eastern site. Between 2009 and 2012, we collected blood samples from an additional 316 song sparrows captured at Newboro (the eastern site) and other sites within 50 km (electronic supplementary material, table S1). Cytochrome *b* was amplified and sequenced as described above.

Of the 350 song sparrows screened (i.e. 34 used in the present study plus 316 in the expanded survey), 53 were infected with *Plasmodium* spp. In all, we identified 11 unique *Plasmodium* lineages (P-SOSP1–P-SOSP11), defined as sequences that differed by at least 1 bp (electronic supplementary material, figure S1). These lineages showed 96–99% sequence identity to other published *Plasmodium* lineages and have been deposited in GenBank (accession numbers KT193627–KT193637).

Several lineages were observed at both the eastern and the western site, but lineage P-SOSP10 (99% sequence identity to morphospecies *P. homopolare*) was detected only at the western site and not at the eastern site or surrounding locations (electronic supplementary material, figure S1 and table S1). Thus, we are confident that this lineage is absent or at least very rare at and around the eastern site, and used it as the ‘western’ lineage in the cross-infection experiment described below. Conversely, lineages P-SOSP9 and P-SOSP11 were detected only at eastern site and not at the western site (electronic supplementary material, figure S1 and table S1). We arbitrarily selected P-SOSP9 (99% nucleotide sequence identity to morphospecies *P. relictum*) as the ‘eastern’ lineage, although as noted above, we cannot exclude the possibility that this strain may also occur in western Ontario. Nucleotide sequence divergence between P-SOSP9 and P-SOSP10 was 8%.

### 2.4. Cross-infection experiment

In October 2012, we collected 200 µl of blood by brachial venipuncture from each of two naturally infected ‘parasite donors’, i.e. an eastern bird infected with P-SOSP9 and a western bird infected with P-SOSP10. Donors showed no evidence of co-infections with other lineages. We used donor blood to inoculate a total of four, previously uninfected, ‘amplifier’ birds, each captured from the same site as its respective donor. We used sympatric rather than allopatric amplifiers to avoid providing parasites with an opportunity to adapt to allopatric hosts. Thus, blood from the eastern donor harbouring P-SOSP9 was injected into two eastern amplifiers, and blood from the western donor harbouring P-SOSP10 was injected into two western amplifiers.

We used a sterile, single-use syringe and 26 gauge needle to inject 200 µl of a mixture containing 50 µl freshly collected (within 5 min) blood, 3.7% sodium citrate and 0.9% saline into the pectoralis muscle of each amplifier over 5–10 s. We monitored the infection status of the four amplifiers by collecting 20 µl blood samples every 3 days between 08.00 and 10.00, and prepared thin-film blood smears as described above. By 18 days after inoculation, all amplifiers showed one to three parasites per 10 000 erythrocytes (average for eastern amplifiers = average for western amplifiers = 2.0 parasites per 10 000 erythrocytes). Asexual (infectious) stages of *Plasmodium* were present in similar concentrations (one to two meronts per 10 000 erythrocytes) in all amplifiers. Amplifiers were euthanized by overdose of isofluorane vapours, and 600 µl of blood immediately collected into a syringe through cardiac puncture. We combined blood from the two eastern amplifiers, and from the two western amplifiers, and mixed each with saline/sodium citrate buffer as described above. Each infected blood mixture was inoculated into ‘experimental’ birds from both the eastern and western site, with 200 µl of infected blood/buffer mixture injected into the pectoralis muscle. ‘Experimental’ birds comprised six eastern birds and six western birds inoculated with the eastern parasite P-SOSP9; and six eastern birds and six western birds inoculated with the western parasite P-SOSP10. As controls, one eastern and one western bird were inoculated with blood from an uninfected eastern bird; and another western bird received no inoculation.

Ten of the experimental birds (five eastern, five western) and one (western) control bird were determined to be naturally infected with *Plasmodium* spp., prior to the start of the experiment. Although none of these naturally occurring infections consisted of P-SOSP9, P-SOSP10 or closely related lineages (5.2% minimum sequence divergence between lineages observed in naturally infected experimental or control birds and lineages P-SOSP9 and P-SOSP10), we included prior infection status as a factor in subsequent analyses.

Beginning 6 days after experimental and control birds were inoculated, we monitored their blood-borne parasite loads every 3 days until 30 days post-inoculation. We collected approximately 20 µl of blood from each bird via brachial venipuncture, between the hours of 08.00 and 10.00. We prepared and screened thin-film blood smears as described above, and scored them blind as regards experimental treatment. Immediately after blood sampling, we also measured each bird's mass to the nearest 0.001 g using a digital scale, and scored subcutaneous furcular fat on a scale of 0 (no visible fat) to 5 (bulging deposits of fat).

### 2.5. Data analysis

In the control bird that tested positive for naturally occurring *Plasmodium* infection, no parasites were detected through microscopy throughout the duration of the experiment. Parasite loads among naturally infected experimental birds prior to the start of the experiment ranged from 0% to 0.02% (mean ± s.e. = 0.40 ± 0.21 parasites per 10 000 erythrocytes). Based on these values, which presumably reflect chronic rather than acute infections, we established an arbitrary threshold for infection success of twice the maximum observed chronic-stage parasitaemia, i.e. 0.04%. Thus, experimentally inoculated birds with at least one observation of at least four infected erythrocytes per 10 000 examined were considered successfully infected. We note that even a single observed gametocyte of the inoculated strain is evidence of successful infection, but because some subjects had previous natural infections, we used a higher threshold to be conservative and to reduce reliance on identifying parasite species through microscopy.

#### 2.5.1. Infection risk

To assess birds' risk of becoming infected with sympatric versus allopatric parasites, we constructed generalized linear model regressions with binomial error distribution using *glm* in R. We used an information-theoretic approach [28] to compare support for eight alternative models (electronic supplementary material, table S2). Each model in the candidate set had infection success as the outcome variable, and included the fixed effects of bird origin (east/west) and parasite origin (east/west). The candidate models differed in the presence versus absence of the interaction term (bird origin × parasite origin) that was of primary interest; previous infection status (i.e. whether or not the subject was already naturally infected with *Plasmodium* upon capture) and sex. We compiled model-averaged parameter estimates from the full set of AICc-ranked candidate models with the natural averaging method [28], using *modavg* in the R package AICcmodavg [29].

#### 2.5.2. Infection severity

For the subset of birds that became infected, we investigated predictors of body mass and fat score using linear-mixed models (*lmer* in the R package lme4 [30]). For each of these dependent variables, we compared support for 16 candidate models (electronic supplementary material, tables S3 and S4) using an information-theoretic approach as described above. Each model in the candidate sets included fixed effects of bird origin and parasite origin, and a random effect of bird ID. Candidate models differed in the presence/absence of the interaction term (bird origin × parasite origin) that was of primary interest; experimental day; previous infection status and sex. We calculated model-averaged parameter estimates as described above.

We used generalized additive mixed models (*gamm* in the R package mgcv [31]) to investigate predictors of parasite load in the subset of birds that became infected. We compared support for 16 candidate models (electronic supplementary material, table S5). All models were fitted with a Poisson distribution and included the fixed effects of bird origin and parasite origin, a random effect of bird ID, and either a single smoother term for experimental day (*k* = 4) or separate smoothers (*k* = 4) for sympatric versus allopatric infections. Candidate models also differed in the presence versus absence of the bird origin × parasite origin interaction term, previous infection status and sex. Models were ranked by AICc and model-averaged parameter estimates calculated using *model.avg* in the R package MuMIn [32].

## 3. Results

### 3.1. Infection risk

**Figure 1.**
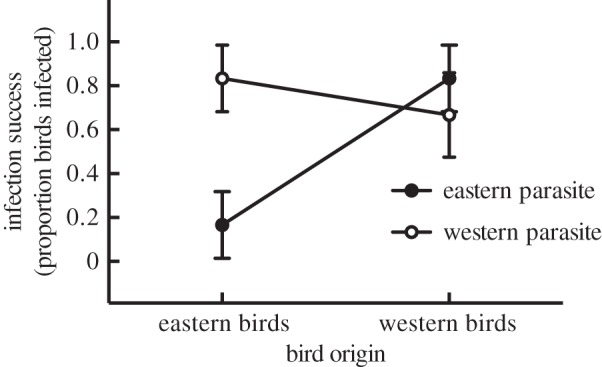
Rates of infection success (defined as one or more observations of at least 0.04% parasitaemia) in song sparrows inoculated with an eastern (P-SOSP9) or western (P-SOSP10) *Plasmodium* lineage. *n* = 24 (6 per group). Data are presented as proportions ± s.e.

Of 24 birds experimentally inoculated with *Plasmodium* spp., all survived to the experimental endpoint of 30 days post-infection, and 15 (63%) became successfully infected. Model selection revealed an interaction between bird origin and parasite origin in predicting infection risk: birds were more likely to become infected when exposed to allopatric parasites than when exposed to sympatric parasites (figure 1). This pattern appears to be driven by low infection success among eastern birds inoculated with the eastern lineage P-SOSP9 (figure 1). Model-averaged confidence intervals for the bird origin × parasite origin interaction term did not overlap with zero (table 1), and models including this interaction were consistently higher-ranked than models that did not (electronic supplementary material, table S2). We found no main effects of bird origin, parasite origin, previous infection status or sex in predicting infection risk (table 1).

**Table 1.** Model-averaged predictors of infection success in 24 song sparrows inoculated with *Plasmodium*. Italic type indicates predictor for which 95% CI does not include zero.

predictor	estimate	s.e.	95% CI (2.5%, 97.5%)
bird origin (western)	1.27	0.97	−0.64, 3.18
parasite origin (western)	−1.36	1.00	−0.61, 3.33
*bird origin* (*western*)* × parasite origin* (*western*)	*−4*.*48*	*2*.*26*	−*8*.*92, −0*.*05*
previous infection status (yes)	−1.87	1.28	−4.38, 0.65
sex (male)	−0.91	1.40	−3.65, 1.83

### 3.2. Infection severity

In contrast to our findings for infection risk, we found no evidence for an interaction between bird origin × parasite origin on body mass or fat score. Model-averaged parameter estimates were not significantly different from zero for any of the candidate predictors of body mass (table 2*a*). Among candidate predictors of fat score, the only variable with a model-averaged parameter estimate significantly different from zero was previous infection status (table 2*b*): birds that had been previously (naturally) infected with *Plasmodium* spp. prior to capture retained more fat than did birds infected for the first time in this study.

**Table 2.** Model-averaged predictors of (*a*) body mass, (*b*) fat score, in 15 song sparrows that became successfully infected. Italic type indicates predictor for which 95% CI does not include zero.

predictor	estimate	s.e.	95% CI (2.5%, 97.5%)
(*a*) body mass			
experimental day	−0.01	0.01	−0.03, 0.01
bird origin (western)	−2.02	1.31	−4.59, 0.55
parasite origin (western)	−1.12	1.32	−3.71, 1.47
bird origin (western) × parasite origin (western)	−3.48	2.84	−9.05, 2.09
previous infection status (yes)	1.48	1.92	−2.29, 5.24
sex (male)	0.69	2.29	−3.79, 5.18
(*b*) fat score			
experimental day	0.01	0.00	0.00, 0.01
bird origin (western)	−0.62	0.41	−1.43, 0.19
parasite origin (western)	−0.61	0.42	−1.44, 0.22
bird origin (western) × parasite origin (western)	−1.41	0.90	−3.18, 0.36
* previous infection status* (*yes*)	*1*.*30*	*0*.*63*	*0*.*06, 2*.*53*
sex (male)	0.47	1.00	−1.49, 2.42

**Figure 2.**
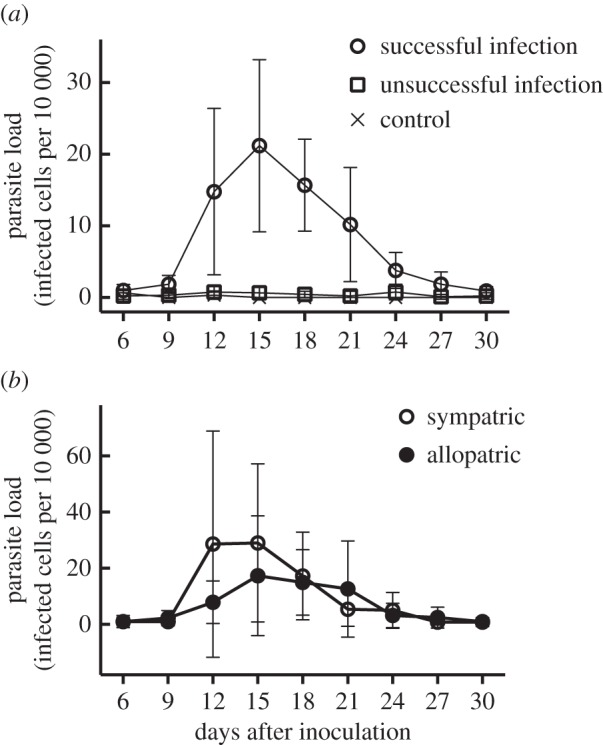
Parasite loads in song sparrows for 30 days after inoculation with *Plasmodium* spp. Values are mean (±95% CI) no. of infected cells per 10 000 erythrocytes. (*a*) Parasite loads in birds categorized as successfully infected (one or more observations of at least 0.04% parasitaemia after inoculation), unsuccessfully infected (inoculated but no observations of at least 0.04% parasitaemia), or control (inoculated with uninfected blood/receiving no inoculation). (*b*) Parasite loads within successfully infected birds, exposed to sympatric (open symbols) or allopatric (filled symbols) *Plasmodium* lineages.

Figure 2*a* shows parasite loads throughout the experiment. Parasitaemia increased then decreased among birds that became successfully infected, in all cases falling below 0.04% by 30 days post-inoculation. Models including separate smoothers for sympatric versus allopatric infections were generally higher-ranked than their counterparts with a single smoother for experimental day (electronic supplementary material, table S5). This pattern suggests differences in the time courses of sympatric versus allopatric infections, with sympatric infections generally showing higher early-stage parasitaemia and reaching peak parasitaemia earlier than allopatric infections (figure 2*b*). However, parasite load was not significantly predicted by the bird origin × parasite origin interaction term, nor did we observe main effects of bird origin, parasite origin, previous infection status or sex on parasite load (table 3).

**Table 3.** Model-averaged predictors of parasite load, in 15 song sparrows that became successfully infected. Smoother terms for experimental day were fitted with *k* = 4. Italic type indicates predictor for which 95% CI does not include zero.

predictor	estimate	s.e.	95% CI (2.5%, 97.5%)
*experimental day smoother* (*1*)	−*1*.*36*	*0*.*55*	−*2*.*44,* −*0*.*29*
*experimental day smoother* (*2*)	*4*.*18*	*0*.*52*	*3*.*15,* *5*.*20*
*experimental day smoother* (*3*)	*1*.*36*	*0*.*63*	*0*.*14,* *2*.*59*
experimental day smoother (1): allopatric	−0.44	0.61	−1.63, 0.76
*experimental day smoother* (*2*)*: allopatric*	*4*.*18*	*0*.*60*	*3*.*01,* *5*.*35*
experimental day smoother (3): allopatric	0.60	0.70	−0.77, 1.96
*experimental day smoother* (*1*)*: sympatric*	−*2*.*77*	*0*.*87*	−*4*.*48,* −*1*.*06*
*experimental day smoother* (*2*)*: sympatric*	*4*.*17*	*0*.*81*	*2*.*59,* *5*.*76*
*experimental day smoother* (*3*)*: sympatric*	*2*.*48*	*0*.*99*	*0*.*53,* *4*.*42*
bird origin (western)	−0.19	0.67	−1.51, 1.12
parasite origin (western)	−0.46	0.68	−1.79, 0.86
bird origin (western) × parasite origin (western)	0.72	1.08	−1.40, 2.85
previous infection status (yes)	0.74	0.56	−0.36, 1.83
sex (male)	−0.27	0.73	−1.70, 1.16

## 4. Discussion

We conducted reciprocal infection experiments to inoculate song sparrows with sympatric or allopatric lineages of *Plasmodium*. Host and parasite origin interacted to predict infection success, a pattern consistent with birds having a home-field advantage in defending against their sympatric parasites. However, the magnitude of this advantage appeared to vary geographically: eastern, but not western, song sparrows were less susceptible to infection by sympatric parasites. Evolutionary and ecological interactions with sympatric haematozoa may, in some cases, provide birds with a parasite-mediated home-field advantage, but the outcome of host–parasite interactions may not be constant over the landscape.

The vast majority of experimental infection studies have used plant or invertebrate hosts [8]. The minority of studies involving vertebrate hosts have yielded mixed findings. In some systems, parasites perform better on sympatric than allopatric hosts, for example in interactions between European minnows (*Phoxinus phoxinus*) and trematodes [33]; black-legged kittiwakes (*Rissa tridactyla*) and ticks [34] and clawed frogs (*Xenopus laevis*) and polystomatid parasites [35]. In other systems, hosts are more resistant to infection by sympatric than allopatric parasites, as in Canarian lizards (*Gallotia galloti*) and their haemogregarine parasites [6], with similar findings in three-spined sticklebacks (*Gasterosteus aculeatus*) infected with trematodes [36]. Meanwhile, in interactions between great tits (*Parus major*) and parasitic fleas, neither hosts nor parasites appear to show local adaptation to the other [37]. One potential reason for the complex patterns observed across vertebrate hosts is that in addition to evolutionary processes (i.e. local arms races with sympatric parasite strains), ecological interactions (i.e. acquired immune memory following exposure to sympatric parasite strains) probably also influence the outcome of host–parasite interactions.

Meta-analyses and theoretical simulations of evolutionary interactions between hosts and parasites identify relative dispersal ability as the key factor that determines whether parasites adapt to their local hosts or hosts to their local parasites [7,8]. Thus, if the typical dispersal distance for song sparrows exceeds that of *Plasmodium* spp., song sparrow populations may become locally adapted to sympatric *Plasmodium* strains. High adult philopatry in these birds [21] suggests that most gene flow results from natal rather than adult dispersal. Natal dispersal distance varies among individuals and populations, but has been estimated at about 6 km in this species [38]. Dispersal of *Plasmodium* spp. occurs passively, through the movements of their invertebrate and vertebrate hosts. Mosquitoes are generally poor fliers [39] and may contribute little to *Plasmodium* dispersal. By contrast, long-distance seasonal migrations of song sparrows and other birds may enhance dispersal distances of haematozoan parasites well beyond those of their hosts [40]. In eastern song sparrows, for example, migratory distances of over 1000 km have been recorded [41]. However, if infected hosts are unable to migrate successfully (migratory culling [42]), then seasonal migration may contribute little to haematozoan dispersal, meaning that the dispersal capacity (and thus adaptive potential) of song sparrow populations could still exceed that of *Plasmodium*. If so, song sparrows may become locally adapted to sympatric *Plasmodium* strains, potentially contributing to the observed home-field advantage of eastern birds in resisting infection by eastern parasites.

Cross-infection experiments represent a significant advance over comparing naturally occurring parasite loads of dispersing versus philopatric individuals, because such experiments are not confounded by variation in individual quality. However, in hosts with the capacity for acquired immune training, reduced susceptibility to sympatric parasites might reflect either host populations being locally adapted to these parasites, and/or host individuals having previously encountered them. This is particularly true when using wild-caught subjects, because previous exposure history is not known. Thus, conclusively disentangling the relative contributions of evolutionary and ecological processes to home-field advantage in dealing with local parasites is beyond the scope of this study. None of the experimental birds in our study were found to be naturally infected with the experimental lineages or with other lineages within 5% sequence similarity, and previous infection by *P. relictum* does not appear to protect against infection by other *Plasmodium* morphospecies [43]. Similarly, in our study previous infection by *Plasmodium* spp. was not significantly associated with infection risk when exposed to the experimental lineages. These observations undermine the role of prior immune experience in this study, and suggest that the home-field advantage we observed may reflect mainly adaptation to local parasites. However, in the light of low sample sizes, as well as the use of only two parasite lineages, we do not rule out prior immune experience as a potential contributor to home-field advantage.

Both microscopy and PCR-based approaches occasionally fail to detect existing infections [44], so we cannot dismiss the possibility that some experimental birds might have previously encountered the experimental lineages or similar variants that could influence their resistance to the strains used in this experiment. Infectivity experiments conducted on wild-caught hosts permit distinguishing home-field advantage from differences in quality between philopatric and dispersing host individuals, but determining whether such advantage results from local adaptation and/or prior exposure will ultimately require exposing immunologically naive hosts to sympatric or allopatric parasites. A recent study accomplished this goal under semi-natural conditions, through transplanting juvenile great tits into outdoor aviaries at sites characterized by different *Plasmodium* communities [45]. Birds of local origin were less likely to become infected by *Plasmodium*, a finding consistent with adaptation by hosts to their local parasites, although the study design did not exclude a role for interactions between birds and biting insect vectors.

The capture sites in our study represent two geographically distinct breeding locations, separated by a distance much greater than the typical natal dispersal distance for song sparrows [38]. However, song sparrows at both locations are seasonally migratory, and the extent of population mixing at the wintering grounds or on migration is uncertain. Haematozoan parasites observed at the breeding grounds could thus have been acquired on the wintering grounds. Although we cannot rule out a wintering-ground origin for the *Plasmodium* lineages used in this study, avian *Plasmodium* can be transmitted as far north as Alaska, at latitudes up to 64°N [46]: thus our breeding-ground capture sites (43–44°N) are well within the latitude at which *Plasmodium* is transmitted. Moreover, parasite donors were captured between July and October, several months after arrival at the breeding grounds, providing considerable opportunity to acquire local parasite lineages. Still, opportunities for adaptation to, and immune training with, the local parasites may be greater for sedentary than migratory hosts, raising the possibility that home-field advantage might be more pronounced among sedentary populations. Similarly, home-field advantage may be stronger when parasites are transmitted directly between conspecific hosts, than when parasites are transmitted by insect vectors [11].

We did not confirm through PCR that successful infections represented the strains of interest. Thus, we cannot conclusively exclude the possibility that some of the observed infections represented a relapse of a previous infection (e.g. owing to the stress of repeated handling and blood sampling) rather than a new, experimentally induced, infection. However, a control individual determined through PCR to be naturally infected at the start of the experiment and subject to the same handling and blood collection regime showed no such relapse; microscopy revealed no parasites in this individual throughout the 30 day duration of the experiment. Still, even if PCR confirmation of the strains infecting experimental birds had been performed, molecular techniques may fail to amplify certain lineages and can thus underestimate the diversity of haematozoan infections within an individual host [47]. Accordingly, we cannot dismiss the possibility that experimental birds may have been co-inoculated with other *Plasmodium* lineages in addition to the intended lineages. Still, co-infections are frequently associated with very high parasite loads and increased mortality [24], whereas we observed relatively low parasitaemia (consistently below 1%), and no mortality even among successfully infected birds. Thus, we think it likely that the experimentally induced infections represent the intended lineages, but alternative interpretations are possible.

Interestingly, the reduced infection risk associated with sympatric parasites was more pronounced for birds of eastern than western origin. Sampling effort was much higher at and around the eastern site than at the western site, thus while we are confident that P-SOSP10 appears to be absent from the eastern site, we cannot dismiss the possibility that P-SOSP9 may also be present at the western site. If so, western song sparrows may have coevolved with and/or previously encountered P-SOSP9 or similar strains, which may explain why these birds had similar risk of infection by their putatively allopatric lineage (P-SOSP9) as by their sympatric lineage (P-SOSP10). Alternatively, because host–parasite interactions vary over space and time, this pattern could reflect local variation in the timing and outcome of antagonistic coevolutionary cycles [48]. Moreover, the two experimental lineages appear to correspond to different morphospecies and may thus differ in virulence, host-specificity, or other factors likely to influence evolutionary arms races. Ecological differences between sites, such as abundance and encounter rates between hosts and parasites, may also contribute to the observed asymmetry of home-field advantage and help to explain why home-field advantage was more pronounced at the eastern site.

Whereas song sparrows from the eastern site were less likely to become infected by sympatric than allopatric parasites, in the subset of birds that became infected we observed no significant difference in the severity of sympatric versus allopatric infections. One limitation of comparing infection severity is that the subset of host individuals that become infected by parasites is not necessarily representative of the wider population in terms of their immune function or condition. In addition, the low numbers of successful sympatric infections observed in this study (*n* = 1 eastern, 4 western) restricts our power to draw conclusions about infection severity. Sympatric infections were associated with somewhat higher parasitaemia during early-stage infections, a pattern that might reflect either a spurious effect of low sample size or an enhanced tolerance of local parasites [49]. Finally, we note that captive studies may not be ideally suited to examine infection severity, because living conditions (handling stress; absence of predators and unrestricted access to food) do not reflect those experienced by free-living animals.

Our findings are relevant to the adaptive significance of dispersal, a key life-history trait with implications for population connectivity. Studies of free-living animals often find that immigrants are in poor condition or have low fitness relative to philopatric individuals [17–19,50], but have not generally been able to distinguish between condition-dependent dispersal and home-field advantage. If sympatric parasites pose less of an infection risk than allopatric parasites, regardless of whether this pattern results from local adaptation and/or prior immune experience with the local parasites, home-field advantage may represent a benefit of philopatry.

Geographical variation in parasites is, of course, only one of many sources of environmental heterogeneity. Thus, the overall fitness consequences of dispersal are likely to be complex. Song sparrow populations in the San Francisco Bay area show adaptations to the local abiotic conditions (i.e. salinity) over a fine geographical scale [51,52]. Among song sparrows on Mandarte Island, meanwhile, male first-generation immigrants are less likely to breed, and female immigrants breed later and lay fewer clutches, relative to birds born on Mandarte, perhaps owing to lack of familiarity with the site [53]. By contrast, performance of immigrants' F_1_ offspring was relatively high (males more likely to breed; earlier breeding for females), consistent with a reduction in inbreeding depression [53]. Collectively, these studies suggest that song sparrows, and probably other animals as well, encounter fine-scale spatial variation in selection pressures. We suggest that infectious disease represents one of many spatially varying selective agents that may influence the fitness consequences of dispersal.

In conclusion, song sparrows were overall less susceptible to infection by sympatric than allopatric malarial parasites, a pattern consistent with home-field advantage to the local parasite strains. This pattern appears to be restricted to birds from the eastern site, consistent with mosaic models of local arms races [9]. Key next steps in disentangling the relative contributions of local adaptation and prior immune experience include cross-infection experiments on hosts known to be immunologically naive, such as birds captured before hatching and hand-raised, together with surveys of geographical variation at candidate immune loci such as major histocompatibility complex (MHC) to identify locally protective alleles. Regardless of the relative contributions of local adaptation and previous immune experience, our findings suggest that parasites may impose fitness costs to dispersal.

## Supplementary Material

Table S1: Sites at which song sparrows were screened for Plasmodium infections. Table S2: AICc-ranked candidate set of models predicting infection success. Table S3: AICc-ranked candidate set of models predicting body mass in the subset of birds that became successfully infected. Table S4: AICc-ranked candidate set of models predicting fat score in the subset of birds that became successfully infected. Table S5: AICc-ranked candidate set of models predicting parasite load in the subset of birds that became successfully infected. Fig. S1. UPGMA phylogeny of 11 cytochrome b lineages of Plasmodium, detected in song sparrows in southeastern and southwestern Ontario; plus 6 similar sequences previously identified to morphospecies.

## Data accessibility

DNA sequences of *Plasmodium* lineages have been deposited to GenBank (accession numbers KT193627-KT193637). Other data supporting this study have been deposited to the Dryad repository: http://dx.doi.org/10.5061/dryad.tk05d.
