# Supplementary material for: Song sparrows Melospiza melodia have a home-field advantage in defending against sympatric malarial parasites
Source: R Soc Open Sci. 2016 Aug 10;3(8):160216. doi: 10.1098/rsos.160216 (PMC5108946; doi:10.1098/rsos.160216)
Supplement: Table S1: Sites at which song sparrows were screened for Plasmodium infections. Table S2: AICc-ranked candidate set of models predicting infection success. Table S3: AICc-ranked candidate set of models predicting body mass in the subset of birds that became successfully infected. Table S4: AICc-rank [file rsos160216supp1.docx]

**Table S1**: Sites at which song sparrows were screened for *Plasmodium* infections. GenBank accession numbers for each *Plasmodium* lineage are provided in Fig. S1. Asterisks denote lineages used for experimental infection.

| Site Name | Coordinates  (°N, °W) | Distance from Newboro (km) | # Birds screened | # Birds infected with *Plasmodium* | *Plasmodium* lineages  (# birds with each lineage) |
| --- | --- | --- | --- | --- | --- |
| Eastern sites | | | | | |
| Newboro | 44.633, 76.330 | -- | 160 | 27 | P-SOSP1 (6)  P-SOSP2 (4)  P-SOSP3 (9)  P-SOSP4 (1)  P-SOSP5 (1)  P-SOSP6 (1)  P-SOSP7 (3)  P-SOSP9* (1)  P-SOSP11 (1) |
| Biology Station | 44.567, 76.324 | 7 | 38 | 2 | P-SOSP3 (2) |
| Swallow Grids | 44.521, 76.385 | 13 | 6 | 2 | P-SOSP3 (2) |
| Murphy’s Point | 44.781, 76.236 | 18 | 20 | 1 | P-SOSP1 (1) |
| Elbow Lake | 44.475, 76.430 | 19 | 21 | 4 | P-SOSP1 (3)  P-SOSP3 (1) |
| Frontenac Park | 44.508, 76.543 | 22 | 21 | 4 | P-SOSP1 (1)  P-SOSP2 (2)  P-SOSP8 (1) |
| Charleston Lake | 44.501, 76.035 | 28 | 19 | 4 | P-SOSP2 (2)  P-SOSP3 (1)  P-SOSP7 (1) |
| Silver Lake | 44.830, 76.579 | 29 | 11 | 3 | P-SOSP1 (1)  P-SOSP2 (1)  P-SOSP3 (1) |
| Sharbot Lake | 44.783, 76.715 | 35 | 4 | 1 | P-SOSP3 (1) |
| Little Cataraqui | 44.289, 76.511 | 41 | 11 | 0 | -- |
| Lemoine Point | 44.226, 76.612 | 51 | 21 | 0 | -- |
| Western site | | | | | |
| London | 43.008, 81.291 | 437 | 19 | 5 | P-SOSP2 (1)  P-SOSP3 (1)  P-SOSP4 (1)  P-SOSP7 (1)  P-SOSP10* (1) |

**Table S2:** Ranked candidate set of models predicting infection success. Independent variables were bird origin (BO), parasite origin (PO), bird origin × parasite origin interaction (BOPO), previous infection status (PREV), and sex. All models were fit with binomial logistic regression.

| Candidate models | K | logLik | AICc | Δ AICc | Weight |
| --- | --- | --- | --- | --- | --- |
| BO + PO + BOPO | 4 | -11.93 | 33.96 | 0 | 0.32 |
| BO + PO + BOPO + PREV | 5 | -10.62 | 34.58 | 0.62 | 0.24 |
| BO + PO | 3 | -14.19 | 35.58 | 1.62 | 0.14 |
| BO + PO + PREV | 4 | -13.18 | 36.46 | 2.49 | 0.09 |
| BO + PO + BOPO + PREV + SEX | 6 | -9.93 | 36.8 | 2.83 | 0.08 |
| BO + PO + BOPO + SEX | 5 | -11.85 | 37.03 | 3.06 | 0.07 |
| BO + PO + SEX | 4 | -14.18 | 38.47 | 4.51 | 0.03 |
| BO + PO + PREV + SEX | 5 | -12.81 | 38.96 | 5.00 | 0.03 |

**Table S3:** Ranked candidate set of models predicting body mass in the subset of birds that became successfully infected. Independent variables were experimental day (DAY), bird origin (BO), parasite origin (PO), bird origin × parasite origin interaction (BOPO), previous infection status (PREV), and sex. All models included bird ID as a random effect.

| Candidate models | K | logLik | AICc | ΔAICc | Weight |
| --- | --- | --- | --- | --- | --- |
| BO + PO | 5 | -211.28 | 433.03 | 0 | 0.16 |
| DAY + BO + PO | 6 | -210.35 | 433.35 | 0.32 | 0.14 |
| BO + PO + BOPO | 6 | -210.5 | 433.66 | 0.63 | 0.12 |
| DAY + BO + PO + BOPO | 7 | -209.56 | 434.01 | 0.98 | 0.1 |
| PREV + BO + PO | 6 | -210.96 | 434.58 | 1.54 | 0.07 |
| DAY + PREV + BO + PO | 7 | -210.02 | 434.93 | 1.9 | 0.06 |
| SEX + BO + PO | 6 | -211.25 | 435.15 | 2.12 | 0.06 |
| DAY + SEX + BO + PO | 7 | -210.31 | 435.5 | 2.47 | 0.05 |
| PREV + BO + PO + BOPO | 7 | -210.35 | 435.58 | 2.54 | 0.04 |
| SEX + BO + PO + BOPO | 7 | -210.5 | 435.88 | 2.85 | 0.04 |
| DAY + PREV + BO + PO + BOPO | 8 | -209.41 | 435.96 | 2.93 | 0.04 |
| SEX + PREV + BO + PO | 7 | -210.57 | 436.02 | 2.98 | 0.04 |
| DAY + SEX + BO + PO + BOPO | 8 | -209.56 | 436.27 | 3.23 | 0.03 |
| DAY + SEX + PREV + BO + PO | 8 | -209.63 | 436.4 | 3.37 | 0.03 |
| SEX + PREV + BO + PO + BOPO | 8 | -210.01 | 437.16 | 4.12 | 0.02 |
| DAY + SEX + PREV + BO + PO + BOPO | 9 | -209.07 | 437.58 | 4.54 | 0.02 |

**Table S4:** Ranked candidate set of models predicting fat score in the subset of birds that became successfully infected. Independent variables were experimental day (DAY), bird origin (BO), parasite origin (PO), bird origin × parasite origin interaction (BOPO), previous infection status (PREV), and sex. All models included bird ID as a random effect.

| Candidate models | K | logLik | AICc | ΔAICc | Weight |
| --- | --- | --- | --- | --- | --- |
| BO + PO + BOPO + PREV | 7 | -135.42 | 285.63 | 0 | 0.16 |
| BO + PO + PREV | 6 | -136.55 | 285.68 | 0.05 | 0.16 |
| DAY + BO + PO + BOPO + PREV | 8 | -134.71 | 286.44 | 0.81 | 0.11 |
| DAY + BO + PO + PREV | 7 | -135.84 | 286.46 | 0.83 | 0.11 |
| BO + PO + PREV + SEX | 7 | -135.99 | 286.77 | 1.14 | 0.09 |
| BO + PO + BOPO + PREV + SEX | 8 | -134.92 | 286.85 | 1.23 | 0.09 |
| DAY + BO + PO + PREV + SEX | 8 | -135.28 | 287.58 | 1.95 | 0.06 |
| DAY + BO + PO + BOPO + PREV + SEX | 9 | -134.21 | 287.7 | 2.07 | 0.06 |
| BO + PO + BOPO + SEX | 7 | -137.12 | 289.03 | 3.41 | 0.03 |
| BO + PO + BOPO | 6 | -138.35 | 289.28 | 3.65 | 0.03 |
| BO + PO + SEX | 6 | -138.47 | 289.53 | 3.90 | 0.02 |
| DAY + BO + PO + BOPO + SEX | 8 | -136.41 | 289.85 | 4.22 | 0.02 |
| DAY + BO + PO + BOPO | 7 | -137.63 | 290.06 | 4.43 | 0.02 |
| BO + PO | 5 | -139.89 | 290.19 | 4.57 | 0.02 |
| DAY + BO + PO + SEX | 7 | -137.76 | 290.31 | 4.68 | 0.02 |

**Table S5:** Ranked candidate set of models predicting parasite load (# *Plasmodium* per 10,000 erythrocytes) in the subset of birds that became successfully infected. Models were generalized additive mixed models fit with Poisson distribution and included bird ID as a random effect. Independent variables included a single smoother term for experimental day (sDAY); separate smoothers for sympatric vs allopatric infections (sDAYTYPE); bird origin (BO); parasite origin (PO); bird origin × parasite origin interaction (BOPO); previous infection status (PREV); and sex.

| Candidate models | K | logLik | AICc | ΔAICc | Weight |
| --- | --- | --- | --- | --- | --- |
| sDAYTYPE + BO + PO | 9 | -241.13 | 501.70 | 0 | 0.291 |
| sDAY + BO + PO | 7 | -244.05 | 502.98 | 1.28 | 0.153 |
| sDAYTYPE + BO + PO + BOPO | 10 | -240.68 | 503.14 | 1.44 | 0.142 |
| sDAY + BO + PO + BOPO | 8 | -243.75 | 504.64 | 2.94 | 0.067 |
| sDAYTYPE + BO + PO + PREV | 10 | -241.53 | 504.84 | 3.14 | 0.061 |
| sDAYTYPE + BO + PO + SEX | 10 | -241.58 | 504.93 | 3.23 | 0.058 |
| sDAYTYPE + BO + PO + BOPO + PREV | 11 | -240.48 | 505.09 | 3.39 | 0.053 |
| sDAYTYPE + BO + PO + BOPO + SEX | 11 | -240.87 | 505.89 | 4.19 | 0.036 |
| sDAY + BO + PO + PREV | 8 | -244.52 | 506.18 | 4.48 | 0.031 |
| sDAY + BO + PO + SEX | 8 | -244.56 | 506.25 | 4.55 | 0.03 |
| sDAYTYPE + BO + PO + PREV + SEX | 11 | -241.38 | 506.90 | 5.20 | 0.022 |
| sDAYTYPE + BO + PO + BOPO + PREV + SEX | 12 | -240.22 | 507.00 | 5.30 | 0.021 |
| sDAY + BO + PO + PREV + SEX | 9 | -244.39 | 508.20 | 6.50 | 0.011 |
| sDAY + BO + PO + BOPO + SEX | 9 | -244.40 | 508.24 | 6.54 | 0.011 |
| sDAY + BO + PO + BOPO + PREV | 9 | -244.47 | 508.38 | 6.68 | 0.01 |
| sDAY + BO + PO + BOPO + PREV + SEX | 10 | -244.26 | 510.29 | 8.59 | 0.004 |

**Fig. S1.** UPGMA phylogeny of 11 cytochrome b lineages of *Plasmodium*, detected in song sparrows in southeastern and southwestern Ontario (GenBank accession numbers reported in parentheses); plus 6 similar sequences previously identified to morphospecies (GenBank accession numbers reported in square brackets). *Haemoproteus tartakovskyi* was used as an outgroup. Arrows denote lineages used for experimental infection (eastern: P-SOSP9, western: P-SOSP10).
